# Influence of Anesthetic Techniques on Colorectal Cancer Recurrence: A Comprehensive Review

**DOI:** 10.7759/cureus.66521

**Published:** 2024-08-09

**Authors:** Sikha Subhadarshini, Karuna Taksande

**Affiliations:** 1 Anaesthesiology, Jawaharlal Nehru Medical College, Datta Meghe Institute of Higher Education and Research, Wardha, IND

**Keywords:** oncological outcomes, perioperative factors, inflammatory response, immune modulation, anesthetic techniques, colorectal cancer recurrence

## Abstract

Colorectal cancer is a leading cause of cancer-related morbidity and mortality worldwide, with a significant risk of recurrence following surgical treatment. Emerging evidence suggests that perioperative factors, particularly anesthetic techniques, may influence cancer recurrence rates. This comprehensive review aims to critically analyze the impact of various anesthetic techniques on colorectal cancer recurrence. We explore the distinct immunomodulatory and inflammatory effects of general, regional, and combined anesthetic approaches and their potential influence on tumor biology. The review synthesizes findings from clinical studies, experimental research, and theoretical models, highlighting the differential impact of anesthetic choices on long-term oncological outcomes. By examining recurrence rates, immune responses, and inflammatory markers associated with different anesthetic techniques, this review provides a holistic understanding of the role of anesthetic management in colorectal cancer surgery. Our findings suggest that anesthetic techniques can modulate the immune and inflammatory responses in ways that may affect tumor recurrence, underscoring the need for further research to optimize anesthetic protocols. The review offers clinical recommendations based on current evidence and identifies gaps in knowledge, proposing directions for future investigations. This comprehensive analysis aims to inform clinical practice and guide future research, ultimately improving long-term outcomes for colorectal cancer patients.

## Introduction and background

Colorectal cancer is one of the most prevalent forms of cancer globally, accounting for a significant portion of cancer-related morbidity and mortality. According to the World Health Organization (WHO), colorectal cancer is the third most commonly diagnosed cancer and the second leading cause of cancer death worldwide [1]. The development of colorectal cancer is a multistep process involving genetic mutations and epigenetic alterations that lead to the transformation of normal colonic epithelium into malignant tumors. Risk factors include age, diet, lifestyle, genetic predisposition, and certain inflammatory bowel conditions [2]. Despite advances in surgical techniques and adjuvant therapies, recurrence of colorectal cancer remains a major clinical challenge. Recurrence rates vary, but it is estimated that approximately 30%-50% of patients experience a recurrence within five years after potentially curative surgery [3]. Understanding the factors that influence recurrence is crucial for improving long-term outcomes. These factors include tumor biology, surgical margins, lymph node involvement, and the systemic inflammatory response. An emerging area of interest is the role of perioperative factors, particularly anesthetic techniques, in influencing cancer recurrence [3].

Anesthetic management is a critical component of colorectal cancer surgeries, affecting not only the immediate perioperative outcomes but also potentially the long-term oncological outcomes. The primary anesthetic techniques used in colorectal cancer surgeries include general anesthesia, regional anesthesia, and combined techniques. General anesthesia involves the use of intravenous and inhalational agents to induce a reversible loss of consciousness and sensation [4]. Regional anesthesia includes epidural and spinal anesthesia, where anesthetic agents are administered near the spinal cord to block sensation in the lower part of the body. Combined techniques often involve general anesthesia supplemented with regional blocks to enhance pain control and reduce opioid requirements. Each of these techniques has distinct pharmacological effects and implications for immune function and inflammatory response, which are believed to influence cancer recurrence [5].

This comprehensive review aims to explore and critically analyze the influence of different anesthetic techniques on the recurrence of colorectal cancer. The review aims to synthesize current evidence from clinical studies, experimental research, and theoretical models to better understand how anesthetic choices made during colorectal cancer surgery can impact long-term oncological outcomes. This review will cover various anesthetic agents' immunomodulatory and inflammatory effects, comparative analyses of recurrence rates associated with different anesthetic techniques, and mechanistic insights into how anesthetic techniques may influence tumor biology. This review seeks to inform clinical practice and guide future research directions in anesthetic management for colorectal cancer patients by addressing these areas.

## Review

Colorectal cancer and recurrence

Epidemiology and Statistics

Colorectal cancer (CRC) represents a major global health issue, as it is the third most commonly diagnosed cancer, with approximately 1.85 million new cases reported annually [1]. This constitutes about 10.2% of all cancers, making it a leading cause of cancer-related morbidity and mortality worldwide. CRC is responsible for nearly one million deaths each year, ranking as the second leading cause of cancer-related deaths. The incidence of colorectal cancer is on the rise, particularly in developing nations adopting Western lifestyles. The contributing factors include obesity, sedentary habits, and dietary choices [6]. Recurrence is a crucial consideration in colorectal cancer management, with studies showing that 30%-40% of patients treated with curative intent experience recurrence within five years. The recurrence rate can vary depending on the cancer stage at diagnosis [7]. For example, earlier studies reported a cumulative recurrence rate of 25.5% for colon cancer, which has decreased to approximately 20.8% in more recent research. Most recurrences occur within the first two to three years following treatment, with distant metastases commonly manifest in these cases [7].

Pathophysiology of Colorectal Cancer Recurrence

The pathophysiology of colorectal cancer recurrence is intricate and involves several mechanisms. One key factor is tumor biology, where the inherent characteristics of the tumor - such as differentiation, molecular markers, and genetic mutations - play a crucial role. Tumors that are poorly differentiated or harbor specific genetic alterations are more likely to recur due to their aggressive nature and potential resistance to treatment [8]. The tumor microenvironment also significantly impacts recurrence. Factors such as inflammatory cells, hypoxia, and nutrient availability can influence tumor growth and metastasis [9]. Additionally, surgical factors are important; incomplete resection or residual microscopic disease can lead to local recurrence. Systemic factors, including the immune response following surgery, are also affected by surgical stress and anesthesia, which may impact the likelihood of recurrence. Immunosuppression from surgical stress or pharmacological agents can enable residual cancer cells to proliferate [9].

Factors Contributing to Recurrence

**Figure 1:**
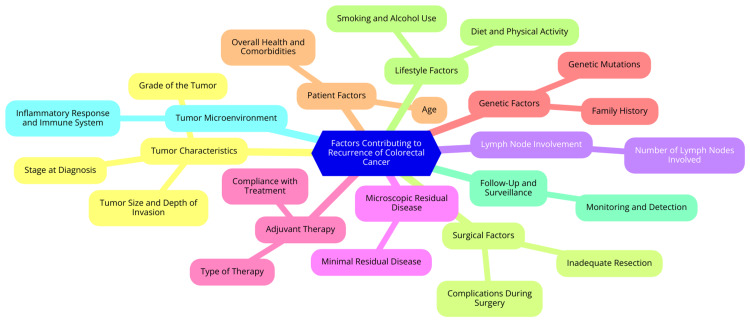
Factors contributing to the recurrence of colorectal cancer Image Credit: Dr Sikha Subhadarshini

Several factors contribute to the recurrence of colorectal cancer, with the tumor stage being one of the most significant. Higher stages, particularly stages II and III, are associated with increased recurrence rates. For instance, patients with stage III disease face a markedly higher risk of recurrence compared to those diagnosed with stage I. Lymph node involvement is another critical predictor; the presence of cancer in lymph nodes suggests a greater likelihood of metastatic spread and subsequent recurrence [10]. The surgical approach also affects recurrence risk. Achieving negative surgical margins is essential for minimizing the chances of local recurrence, as positive margins indicate residual disease. Adjuvant therapy, especially chemotherapy, plays a significant role in lowering recurrence risk, particularly in stage III patients. However, variability in treatment adherence and the effectiveness of different regimens can impact outcomes [11]. Lifestyle factors, including diet, physical activity, smoking, and alcohol consumption, also influence recurrence rates. A diet high in fiber and low in red and processed meats is linked to a reduced risk of recurrence. Moreover, genetic predisposition can contribute to recurrence; patients with hereditary syndromes such as Lynch syndrome are at a higher risk of recurrent disease due to genetic factors that predispose them to multiple malignancies [12]. Factors contributing to the recurrence of colorectal cancer are illustrated in Figure 1.

Anesthetic techniques in colorectal cancer surgery

Anesthetic techniques are pivotal in managing patients undergoing colorectal cancer surgery, as they can impact both immediate postoperative recovery and long-term outcomes, including cancer recurrence. The main anesthetic approaches are general, regional, and combined techniques, each with specific mechanisms, agents, and benefits [4]. General anesthesia induces a reversible loss of consciousness and sensation, facilitating the safe performance of extensive surgical procedures. Although the exact mechanisms are not fully understood, general anesthetics are believed to primarily act on various receptors in the central nervous system (CNS) [13]. Key mechanisms include enhancing gamma-aminobutyric acid (GABA) receptor activity, which hyperpolarizes neurons and leads to sedation and unconsciousness. Some agents, such as ketamine, also block N-methyl-D-aspartate (NMDA) receptors, contributing to analgesic effects. Common agents used in general anesthesia include intravenous agents like propofol, favored for its rapid onset and recovery, and inhalational agents such as isoflurane and sevoflurane, known for their effectiveness in maintaining anesthesia during surgery [14]. Regional anesthesia involves administering anesthetic agents to specific body regions, providing targeted pain relief without affecting consciousness. Epidural anesthesia, a common technique, involves injecting anesthetic into the epidural space surrounding the spinal cord, effectively blocking nerve signals in the lower body. This method is beneficial for managing pain during and after surgery, as it reduces the need for systemic opioids and can enhance postoperative recovery [5]. Another approach, spinal anesthesia, involves injecting anesthetic directly into the cerebrospinal fluid in the spinal canal, resulting in the rapid onset of anesthesia in the lower body. This technique is useful for surgeries below the umbilicus, offering profound muscle relaxation and pain relief. Additionally, nerve blocks can be utilized, where local anesthetics are injected near specific nerves to block sensation in targeted areas, such as the femoral nerve or lumbar plexus. This localized pain relief can further enhance recovery and reduce systemic opioid use [15]. Combined techniques incorporating general and regional anesthesia are increasingly used to optimize pain management and recovery. This approach allows for reduced opioid consumption, as regional blocks can significantly decrease the number of opioids needed during and after surgery. Consequently, this minimizes side effects such as nausea and sedation, leading to an overall improved recovery profile. Patients often experience quicker recovery of bowel function and reduced postoperative pain, which can result in shorter hospital stays [16].

Impact of anesthetic techniques on immune function

The impact of anesthetic techniques on immune function is a critical area of research, especially about cancer recurrence. Anesthetic agents can modulate immune responses, influencing both innate and adaptive immunity, which may have significant implications for patients undergoing cancer surgery. The immunomodulatory effects of anesthesia are complex and can vary depending on the anesthetic used [17]. General anesthesia can alter cytokine production and immune cell activity, particularly when administered over extended periods. While these effects are generally minimal in healthy patients undergoing brief procedures, significant changes are often attributed to surgical trauma rather than the anesthesia itself. Volatile anesthetics, such as sevoflurane and isoflurane, have been shown to exert both immunosuppressive and immunoactivating effects [18]. For instance, these agents may negatively impact anti-tumor immunity. The effects of propofol are less clear, with some studies suggesting it may offer potential benefits. Local anesthetics can help alleviate surgical stress responses and may enhance immune function by blocking nerve signals that activate the hypothalamic-pituitary-adrenal (HPA) axis, known for its immunosuppressive effects. Additionally, local anesthetics might directly affect the activity of natural killer (NK) cells, although the outcomes can vary based on concentration [19]. The immune system is crucial for recognizing and eliminating cancer cells, making its function essential in the context of cancer recurrence. Anesthetic-induced immunomodulation can significantly influence recurrence rates. Immunosuppression resulting from anesthesia may facilitate tumor growth and metastasis, particularly in patients with existing malignancies [20]. Activation of the HPA axis and the sympathetic nervous system during surgery can lead to the release of immunosuppressive cytokines that impair the body’s ability to combat tumor cells. Conversely, certain anesthetic techniques that enhance immune function could potentially reduce the risk of cancer recurrence. For example, local anesthetics may support immune responses by mitigating the stress response associated with surgery [21]. Comparative analyses of immune responses under different anesthetic techniques reveal that these techniques produce varying effects on immune function, which can impact patient outcomes. Studies suggest that inhalational anesthetics may have more pronounced immunosuppressive effects than intravenous agents like propofol. This discrepancy underscores the importance of carefully selecting anesthetic techniques based on the patient’s immune status and the type of surgery. Regional anesthesia techniques, such as epidural anesthesia, have been proposed to provide benefits by reducing surgical stress and preserving immune function. However, evidence regarding their impact on cancer recurrence remains inconclusive, warranting further investigation [22].

Influence of anesthetic techniques on inflammatory response

The influence of anesthetic techniques on the inflammatory response is a crucial area of research, especially regarding their potential impact on cancer recurrence. Surgical trauma triggers a systemic inflammatory response that can affect cancer recurrence. The magnitude of this inflammatory response is linked to postoperative outcomes, including the risk of metastasis. Anesthetic techniques may modulate this response, potentially affecting the immune system's ability to target residual cancer cells [23]. General anesthesia (GA) has been shown to have varying effects on inflammatory markers. For example, GA can increase pro-inflammatory cytokines, which may contribute to postoperative complications. However, specific anesthetic agents, such as propofol, have been associated with lower levels of inflammatory markers like C-reactive protein (CRP) and interleukin-6 (IL-6) compared to inhalational agents [24]. Furthermore, combining GA with epidural anesthesia has been reported to attenuate the increase in inflammatory mediators, potentially leading to improved postoperative immune function and better outcomes. The immunomodulatory effects of anesthetics, including their ability to inhibit the stress response and reduce catecholamine release, may contribute to this process [25]. Regional anesthesia techniques, such as epidural and nerve blocks, are believed to benefit the inflammatory response. These techniques may reduce the surgical stress response and the need for systemic opioids, further decreasing inflammation. Studies suggest that regional anesthesia can better preserve immune function than general anesthesia alone, potentially lowering the risk of cancer recurrence by enhancing immune surveillance against tumor cells [26]. The choice of anesthetic technique significantly affects perioperative inflammatory markers. Research indicates that total intravenous anesthesia (TIVA) using propofol is associated with lower levels of inflammatory markers compared to inhalational anesthetics. In contrast, volatile anesthetics like sevoflurane may promote inflammatory pathways, potentially increasing the risk of cancer recurrence [27].

Pain management and cancer recurrence

Effective pain management is crucial in cancer care as it significantly impacts patient quality of life and may influence cancer recurrence. The relationship between pain management strategies, particularly the use of opioids and non-opioid analgesics, and cancer recurrence is complex and requires careful consideration [28]. Effective pain management is essential for improving the quality of life in cancer patients. Uncontrolled pain can lead to decreased functional status and psychological distress, making optimal pain control a priority both during the perioperative period and throughout treatment. Multimodal analgesia, which combines various analgesic methods, is increasingly recognized for its role in minimizing opioid use and associated side effects [29]. Opioids are commonly prescribed for moderate to severe cancer-related pain. However, their role in cancer recurrence is controversial. Opioids may impair immune function, potentially facilitating tumor growth and metastasis. Some studies suggest that opioid receptor activation can lead to immunosuppression and increased angiogenesis, which might promote cancer progression [30]. Despite these concerns, clinical studies have produced mixed results. While some retrospective studies indicate a possible association between opioid use during surgery and higher rates of cancer recurrence, others find no significant correlation. A systematic review focusing on breast cancer found no clear link between opioid use and recurrence rates, underscoring the need for further research [31]. Non-opioid analgesics, including nonsteroidal anti-inflammatory drugs (NSAIDs) and cyclooxygenase 2 (COX-2) inhibitors, are being investigated for their potential benefits in cancer pain management and recurrence prevention. Certain non-opioid analgesics, such as celecoxib, have shown promise in reducing tumor growth and improving postoperative outcomes in some studies. They may also counteract the proangiogenic effects of opioids, suggesting a beneficial role in cancer management [32]. The use of multimodal analgesia, which includes non-opioid medications, is encouraged to reduce opioid consumption and potentially improve cancer outcomes. This approach aims to harness the benefits of various analgesics while minimizing the risks associated with opioids [32]. Regional anesthesia techniques, such as epidural or nerve blocks, can significantly decrease the need for opioids during and after surgery. By providing effective pain relief, regional anesthesia can reduce opioid consumption, which may help mitigate the adverse effects associated with opioid use, including immune suppression [33]. Some studies suggest that regional anesthesia might be linked to improved cancer outcomes, potentially due to reduced surgical stress responses and preserved immune function. However, results have been inconsistent, and more rigorous research is needed to establish these associations conclusively [33].

Clinical evidence and studies

The influence of anesthetic techniques on colorectal cancer recurrence has been extensively studied, with research focusing on the comparative effects of general anesthesia (GA) and regional anesthesia (RA) and the impact of specific anesthetic agents. A substantial body of clinical evidence suggests that the choice of anesthetic technique can significantly affect patient outcomes following colorectal surgery [34]. Several studies have directly compared GA and RA in the context of cancer recurrence. For example, randomized controlled trials have shown that patients receiving RA, including techniques such as epidural analgesia, may experience improved disease-free survival rates compared to those undergoing GA alone. This observation supports the hypothesis that RA may mitigate the surgical stress response and enhance immune function, potentially reducing cancer recurrence [35]. A multicenter controlled trial further demonstrated that epidural analgesia could result in better disease-free survival outcomes after colorectal surgery compared to intravenous analgesia [36]. The choice of specific anesthetic agents also appears to influence recurrence rates. Research comparing inhalational anesthetics, such as sevoflurane, with intravenous agents like propofol has revealed notable differences in outcomes. Inhalational agents have been linked to increased cancer cell proliferation and metastasis in preclinical studies, while propofol has shown potential protective effects against these pathways [37]. Systematic reviews have highlighted that patients receiving propofol-based anesthesia often exhibit lower recurrence rates compared to those treated with sevoflurane, emphasizing the importance of anesthetic choice in oncological settings [38]. Meta-analyses have synthesized findings from multiple studies, consistently indicating that RA or total intravenous anesthesia (TIVA) may lead to better oncological outcomes than traditional GA. These reviews underscore the need for further large-scale, randomized trials to confirm these observations and refine clinical practices in anesthetic management for colorectal cancer surgeries [39]. In addition to randomized trials, retrospective studies have provided valuable insights into the relationship between anesthetic techniques and cancer recurrence. Comprehensive reviews of such studies have consistently found a link between RA and improved overall survival (OS) and recurrence-free survival (RFS) in colorectal cancer patients. These analyses often emphasize the role of anesthetic techniques in modulating immune responses, which could significantly influence cancer recurrence rates [34].

Mechanisms linking anesthetic techniques to recurrence

The study of colorectal cancer provides a valuable framework for understanding disease susceptibility and the somatic evolution of epithelial cancers. Theoretical models, especially those utilizing animal studies, are essential for investigating colon cancer progression. These models enable researchers to track tumor development and responses to various interventions, including different anesthetic techniques. Such approaches are crucial for uncovering new prevention strategies and treatment modalities, offering insights into how anesthetic choices can impact cancer outcomes [40]. At the molecular and cellular levels, anesthetic techniques significantly modulate immune responses, critical for cancer recurrence. Regional anesthesia (RA), such as epidural or spinal anesthesia, has been shown to preserve immune function better than general anesthesia (GA). This preservation is important as it maintains higher activity levels of natural killer (NK) and T cells, vital for anti-tumor immunity [41]. Additionally, RA is associated with a reduced surgical stress response, leading to a lower release of pro-inflammatory cytokines that can promote tumor progression. In contrast, certain inhalational anesthetics like sevoflurane have been linked to increased cancer cell proliferation and angiogenesis. In contrast, intravenous agents such as propofol may offer protective effects against these adverse outcomes [42]. The choice of anesthetic agents also influences the tumor microenvironment, which is crucial for cancer recurrence. Inhalational anesthetics may create conditions that support tumor growth, while intravenous anesthetics like propofol are associated with anti-inflammatory properties that might mitigate such risks. Furthermore, RA techniques can reduce opioid requirements post-surgery [43]. High opioid use has been linked to immunosuppression and adverse outcomes in cancer patients, including increased recurrence rates. By minimizing opioid consumption, RA may help maintain immune function and create a more favorable environment for the immune system to combat residual cancer cells after surgery. Moreover, RA is generally associated with less postoperative pain and quicker recovery, allowing for the earlier initiation of adjuvant therapies, which is crucial for improving long-term outcomes in colorectal cancer patients [34].

Recommendations for clinical practice

Current guidelines and best practices in anesthetic management, particularly concerning their impact on cancer recurrence, emphasize a personalized approach to anesthetic choice based on evidence and patient-specific factors. The American Society of Anesthesiologists (ASA) provides practice guidelines to help practitioners make informed decisions about anesthesia care. These guidelines are based on systematic reviews of literature and expert consensus, ensuring they stay relevant as new evidence emerges. They stress the importance of individualized care tailored to each patient's unique needs and clinical circumstances [44]. Similarly, the Canadian Anesthesia Society has updated its guidelines to include specific recommendations for managing controlled substances, fasting protocols, and neuromuscular monitoring. These guidelines aim to enhance patient safety and improve outcomes by providing clear protocols for anesthetic practice. Additionally, updated guidelines on difficult airway management focus on strategies and equipment necessary for effective airway management during anesthesia, highlighting the importance of preparedness and algorithms to assist practitioners in challenging situations [45].

Evidence suggests that regional anesthesia may offer beneficial effects on oncological outcomes by reducing the stress response and minimizing opioid requirements. This approach is particularly relevant for colorectal surgery patients, as it may help mitigate potential risks associated with general anesthesia. Additionally, the use of total intravenous anesthesia (TIVA) with agents like propofol has been linked to lower rates of tumor recurrence compared to inhalational anesthetics. Ongoing clinical trials actively investigate the long-term outcomes of various anesthetic techniques on cancer recurrence, which will provide further insights into this crucial issue [46]. A personalized anesthetic approach is essential for optimizing patient outcomes. Anesthetic choices should be tailored to individual patient profiles, considering factors such as medical history, type of surgery, and specific oncological considerations. This individualized strategy ensures that the selected anesthetic technique aligns with the patient’s overall treatment goals and enhances recovery [47].

Assessing patient-specific factors is crucial for clinicians when selecting anesthetic techniques. Factors such as comorbidities, cancer type, and surgical complexity must be evaluated to choose the most appropriate anesthetic strategy that balances efficacy with safety. Incorporating patient preferences into decision-making can also enhance satisfaction and adherence to treatment plans. Engaging patients in discussions about their anesthetic options and addressing their concerns regarding anesthesia and recovery can significantly improve their overall experience [48]. Continuous monitoring of patient responses during the perioperative period is essential for making necessary adjustments in anesthetic management. This adaptability is critical for optimizing outcomes and minimizing complications. In summary, current guidelines advocate for a personalized approach to anesthetic management, emphasizing the importance of evidence-based practices while considering individual patient needs and preferences. Ongoing research will continue to refine these recommendations as more data becomes available regarding the impact of anesthetic techniques on cancer recurrence [49].

Future research directions

Despite growing interest in the relationship between anesthetic techniques and colorectal cancer recurrence, several critical gaps in current knowledge persist. One major area requiring further exploration is the underlying biological mechanisms by which different anesthetics influence tumor behavior and immune response. While some studies indicate that certain anesthetics may promote or inhibit cancer cell proliferation, the specific pathways involved remain poorly understood [34]. Additionally, many existing studies are retrospective and lack long-term follow-up data, making it difficult to draw definitive conclusions about the long-term effects of anesthetic choices on cancer recurrence and overall survival. Variability in anesthetic protocols across different studies further complicates comparisons and hinders the establishment of standardized practices. Finally, individual patient factors, such as genetic predispositions and comorbidities, may significantly affect the efficacy of anesthetic techniques, yet research on these interactions is still limited [50].

Several emerging techniques and technologies have shown promise in optimizing anesthetic management for colorectal cancer surgeries in recent years. Enhanced recovery after surgery (ERAS) protocols are gaining traction, aiming to improve perioperative care through a multidisciplinary approach that includes specific anesthetic strategies [51]. Total intravenous anesthesia (TIVA), particularly with agents like propofol, is being investigated for its potential advantages over traditional inhalational anesthesia, especially regarding immune modulation and cancer recurrence. Intravenous lidocaine is also emerging as an adjunct to anesthesia, with preliminary studies suggesting it may reduce postoperative pain and potentially influence cancer outcomes favorably. Additionally, advances in molecular biology are paving the way for biomarker research, which could help identify patients who may benefit most from specific anesthetic techniques based on their tumor characteristics [52].

Several key areas for future investigation should be prioritized to address the existing gaps in knowledge. First, mechanistic studies are essential to elucidate the cellular and molecular pathways through which anesthetics impact cancer biology, including their effects on apoptosis, cell migration, and immune system responses during surgery. Comparative effectiveness research is also crucial; more studies should focus on comparing the outcomes of different anesthetic techniques, particularly examining the benefits of TIVA versus inhalational anesthesia regarding recurrence rates and quality of life [53]. Conducting multicenter trials would help gather a larger and more diverse patient population, enhancing the generalizability of findings. Furthermore, exploring how anesthetic techniques can be integrated with other cancer therapies, such as chemotherapy and immunotherapy, may yield valuable insights into comprehensive treatment strategies that minimize recurrence. Lastly, future studies should emphasize patient-centered outcomes, including quality of life and functional recovery, alongside traditional clinical endpoints like recurrence and survival rates. By focusing on these areas, future research can significantly advance our understanding of the interplay between anesthetic techniques and colorectal cancer recurrence, ultimately leading to improved patient care [54].

## Conclusions

In conclusion, the choice of anesthetic technique during colorectal cancer surgery appears to have significant implications for long-term oncological outcomes, particularly cancer recurrence. General anesthesia, regional anesthesia, and combined techniques have distinct effects on immune function and inflammatory responses, which are crucial factors in tumor progression and recurrence. Evidence from clinical studies and experimental research suggests that regional anesthesia and combined techniques may offer advantages in reducing recurrence rates compared to general anesthesia alone, likely due to their role in preserving immune function and minimizing systemic inflammation. However, the current body of evidence is still evolving, and more high-quality, large-scale studies are needed to definitively establish the impact of anesthetic techniques on colorectal cancer recurrence. Clinicians should consider these findings when planning perioperative care and weigh the potential benefits of different anesthetic approaches. Future research should continue to explore the underlying mechanisms and optimize anesthetic strategies to improve long-term outcomes for colorectal cancer patients.

## References

[1] (2024). Colorectal cancer. https://www.who.int/news-room/fact-sheets/detail/colorectal-cancer.

[2] Armaghany T, Wilson JD, Chu Q, Mills G (2012). Genetic alterations in colorectal cancer. Gastrointest Cancer Res.

[3] Ryu HS, Kim J, Park YR (2023). Recurrence patterns and risk factors after curative resection for colorectal cancer: insights for postoperative surveillance strategies. Cancers (Basel).

[4] Ramirez MF, Cata JP (2021). Anesthesia techniques and long-term oncological outcomes. Front Oncol.

[5] Folino TB, Mahboobi SK (2024). Regional anesthetic blocks. StatPearls.

[6] Rawla P, Sunkara T, Barsouk A (2019). Epidemiology of colorectal cancer: incidence, mortality, survival, and risk factors. Prz Gastroenterol.

[7] Nors J, Iversen LH, Erichsen R, Gotschalck KA, Andersen CL (2024). Incidence of recurrence and time to recurrence in stage I to III colorectal cancer: a nationwide danish cohort study. JAMA Oncol.

[8] Kudryavtseva AV, Lipatova AV, Zaretsky AR (2016). Important molecular genetic markers of colorectal cancer. Oncotarget.

[9] Li Y, Zhao L, Li XF (2021). Hypoxia and the tumor microenvironment. Technol Cancer Res Treat.

[10] Xiong ZZ, Xie MH, Li XZ (2023). Risk factors for postoperative recurrence in patients with stage II colorectal cancer. BMC Cancer.

[11] Houssami N, Macaskill P, Marinovich ML, Morrow M (2014). The association of surgical margins and local recurrence in women with early-stage invasive breast cancer treated with breast-conserving therapy: a meta-analysis. Ann Surg Oncol.

[12] Anand P, Kunnumakkara AB, Sundaram C (2008). Cancer is a preventable disease that requires major lifestyle changes. Pharm Res.

[13] Siddiqui BA, Kim PY (2023). Anesthesia stages. StatPearls.

[14] Allen MJ, Sabir S, Sharma S (2023). GABA receptor. StatPearls.

[15] Olawin AM, Das JM (2022). Spinal anesthesia. StatPearls.

[16] Ferry N, Hancock LE, Dhanjal S (2023). Opioid anesthesia. StatPearls.

[17] Brogi E, Forfori F (2022). Anesthesia and cancer recurrence: an overview. J Anesth Analg Crit Care.

[18] Jafarzadeh A, Hadavi M, Hassanshahi G, Rezaeian M, Vazirinejad R (2020). General anesthetics on immune system cytokines: a narrative review article. Anesth Pain Med.

[19] Luan T, Li Y, Sun L, Xu S, Wang H, Wang J, Li C (2022). Systemic immune effects of anesthetics and their intracellular targets in tumors. Front Med (Lausanne).

[20] Gonzalez H, Hagerling C, Werb Z (2018). Roles of the immune system in cancer: from tumor initiation to metastatic progression. Genes Dev.

[21] Kim R (2018). Effects of surgery and anesthetic choice on immunosuppression and cancer recurrence. J Transl Med.

[22] Konstantis G, Tsaousi G, Kitsikidou E, Zacharoulis D, Pourzitaki C (2023). The immunomodulatory effect of various anaesthetic practices in patients undergoing gastric or colon cancer surgery: a systematic review and meta-analysis of randomized clinical trials. J Clin Med.

[23] Forget P, Simonet O, De Kock M (2013). Cancer surgery induces inflammation, immunosuppression and neo-angiogenesis, but is it influenced by analgesics?. F1000Res.

[24] Alhayyan A, McSorley S, Roxburgh C, Kearns R, Horgan P, McMillan D (2020). The effect of anesthesia on the postoperative systemic inflammatory response in patients undergoing surgery: a systematic review and meta-analysis. Surg Open Sci.

[25] Zhang Y, Lu J, Qin M (2023). Effects of different anesthesia methods on postoperative immune function in patients undergoing gastrointestinal tumor resection. Sci Rep.

[26] Kianian S, Bansal J, Lee C, Zhang K, Bergese SD (2024). Perioperative multimodal analgesia: a review of efficacy and safety of the treatment options. Anesthesiol Perioper Sci.

[27] Ștefan M, Predoi C, Goicea R, Filipescu D (2022). Volatile anaesthesia versus total intravenous anaesthesia for cardiac surgery - a narrative review. J Clin Med.

[28] Scarborough BM, Smith CB (2018). Optimal pain management for patients with cancer in the modern era. CA Cancer J Clin.

[29] Mestdagh F, Steyaert A, Lavand'homme P (2023). Cancer pain management: a narrative review of current concepts, strategies, and techniques. Curr Oncol.

[30] Lucia M, Luca T, Federica DP (2021). Opioids and breast cancer recurrence: a systematic review. Cancers (Basel).

[31] National Academies of Sciences, Engineering Engineering, and Medicine; Health and Medicine Division; Board on Health Sciences Policy; Committee on Pain Management and Regulatory Strategies to Address Prescription Opioid Abuse (2017). Evidence on strategies for addressing the opioid epidemic. Pain Management and the Opioid Epidemic: Balancing Societal and Individual Benefits and Risks of Prescription Opioid Use.

[32] Magee DJ, Jhanji S, Poulogiannis G, Farquhar-Smith P, Brown MR (2019). Nonsteroidal anti-inflammatory drugs and pain in cancer patients: a systematic review and reappraisal of the evidence. Br J Anaesth.

[33] (2024). Regional anesthesia for postoperative pain control in orthopedic surgery: overview, neuraxial analgesia, peripheral nerve blocks. Published Online First: 12 July.

[34] Xia SH, Zhou D, Ge F, Sun M, Chen X, Zhang H, Miao C (2023). Influence of perioperative anesthesia on cancer recurrence: from basic science to clinical practice. Curr Oncol Rep.

[35] Xie S, Li L, Meng F, Wang H (2024). Regional anesthesia might reduce recurrence and metastasis rates in adult patients with cancers after surgery: a meta-analysis. BMC Anesthesiol.

[36] Falk W, Magnuson A, Eintrei C, Henningsson R, Myrelid P, Matthiessen P, Gupta A (2021). Comparison between epidural and intravenous analgesia effects on disease-free survival after colorectal cancer surgery: a randomised multicentre controlled trial. Br J Anaesth.

[37] Enlund M, Berglund A, Andreasson K, Cicek C, Enlund A, Bergkvist L (2014). The choice of anaesthetic - sevoflurane or propofol - and outcome from cancer surgery: a retrospective analysis. Ups J Med Sci.

[38] Lee S, Pyo DH, Sim WS, Lee WY, Park M (2022). Early and long-term outcomes after propofol-and sevoflurane-based anesthesia in colorectal cancer surgery: a retrospective study. J Clin Med.

[39] Yap A, Lopez-Olivo MA, Dubowitz J, Hiller J, Riedel B (2019). Anesthetic technique and cancer outcomes: a meta-analysis of total intravenous versus volatile anesthesia. Can J Anaesth.

[40] Neto Í, Rocha J, Gaspar MM, Reis CP (2023). Experimental murine models for colorectal cancer research. Cancers (Basel).

[41] Cata JP, Guerra C, Soto G, Ramirez MF (2020). Anesthesia options and the recurrence of cancer: what we know so far?. Local Reg Anesth.

[42] Jing Y, Zhang Y, Pan R, Ding K, Chen R, Meng Q (2022). Effect of inhalation anesthetics on tumor metastasis. Technol Cancer Res Treat.

[43] Yang W, Cai J, Zabkiewicz C, Zhang H, Ruge F, Jiang WG (2017). The effects of anesthetics on recurrence and metastasis of cancer, and clinical implications. World J Oncol.

[44] Liu X, Wang Q (2022). Application of anesthetics in cancer patients: reviewing current existing link with tumor recurrence. Front Oncol.

[45] Thilen SR, Weigel WA, Todd MM (2023). 2023 American Society of Anesthesiologists Practice Guidelines for Monitoring and Antagonism of Neuromuscular Blockade: A Report by the American Society of Anesthesiologists Task Force on Neuromuscular Blockade. Anesthesiology.

[46] Hutton M, Brull R, Macfarlane AJ (2018). Regional anaesthesia and outcomes. BJA Educ.

[47] Smith G, D’Cruz JR, Rondeau B, Goldman J (2023). General anesthesia for surgeons. StatPearls.

[48] Zambouri A (2007). Preoperative evaluation and preparation for anesthesia and surgery. Hippokratia.

[49] Tippireddy S, Ghatol D (2023). Anesthetic management for enhanced recovery after major surgery (ERAS). StatPearls.

[50] Dubowitz J, Ziegler AI, Beare R (2023). Type of anesthesia for cancer resection surgery: no differential impact on cancer recurrence in mouse models of breast cancer. PLoS One.

[51] Turaga AH (2023). Enhanced recovery after surgery (ERAS) protocols for improving outcomes for patients undergoing major colorectal surgery. Cureus.

[52] Seo KH, Hong JH, Moon MH (2023). Effect of total intravenous versus inhalation anesthesia on long-term oncological outcomes in patients undergoing curative resection for early-stage non-small cell lung cancer: a retrospective cohort study. Korean J Anesthesiol.

[53] Stollings LM, Jia LJ, Tang P, Dou H, Lu B, Xu Y (2016). Immune modulation by volatile anesthetics. Anesthesiology.

[54] Patil DJ, Vyas T, Kataria AP, Rajput R, Ashem A, Kumar M (2023). Multi-centric clinic trials in evidence-based research - a narrative review on the Indian scenario. J Family Med Prim Care.

